# Novel DNA Aptamers to Dickkopf-1 Protein and Their Application in Colorimetric Sandwich Assays for Target Detection in Patients with Axial Spondyloarthritis

**DOI:** 10.3390/ijms252212214

**Published:** 2024-11-14

**Authors:** Elizaveta A. Shatunova, Anastasia S. Rychkova, Mariya I. Meschaninova, Marsel R. Kabilov, Alexey E. Tupikin, Yuliya D. Kurochkina, Maksim A. Korolev, Mariya A. Vorobyeva

**Affiliations:** 1 Institute of Chemical Biology and Fundamental Medicine, Siberian Division of the Russian Academy of Sciences, Lavrentiev Ave. 8, Novosibirsk 630090, Russia; lizashatunova@yandex.ru (E.A.S.); mesch@niboch.nsc.ru (M.I.M.); kabilov@niboch.nsc.ru (M.R.K.); kormax@bk.ru (M.A.K.); 2 Research Institute of Clinical and Experimental Lymphology, Affiliated Branch of Federal Research Center of Cytology and Genetics, Siberian Division of the Russian Academy of Sciences, Timakova St. 2, Novosibirsk 630060, Russia

**Keywords:** Dickkopf-1 protein, DKK-1, aptamers, in vitro selection, ankylosing spondylitis, Bekhterev’s disease, colorimetric detection

## Abstract

Chronic immunoinflammatory rheumatic diseases, such as axial spondyloarthritis (AxSpA), are accompanied by a dysregulation of bone remodeling. Among potential biomarkers of bone metabolism, the Wnt pathway antagonist, Dickkopf-1 (DKK-1), is of particular interest because of its potential to reflect a shift towards joint ossification or osteoporosis, but its diagnostic value needs validation. There is still a lack of stable and efficient methods of measuring serum DKK-1 levels suitable for longitude studies. The use of aptamer-based diagnostic assays could be very promising for this purpose. We generated novel anti-DKK-1 DNA aptamers from a combinatorial library with a pre-defined sequence pattern in the randomized region. This approach showed high efficacy, as only four SELEX rounds of selection produced high-affinity aptamers with dissociation constants ranging from 1.3 to 3.7 nM. A family of their truncated versions was also developed by rational design. Novel DNA aptamers functioned as capture components in a microplate ELISA-like assay with HRP-conjugated anti-DKK-1 antibody as a reporter component. We succeeded in revealing the aptamer/aptamer sandwich pairs that provided an aptamer-only sandwich colorimetric assay. The aptamer/antibody colorimetric test systems were also examined in the analyses of blood serum from AxSpA patients and shown sufficient workability. However, in a number of cases we registered significant differences between assays based on TD10 and DK4 aptamers and made some suggestions about the origin of this effect.

## 1. Introduction

Axial spondyloarthritis (AxSpA) is a chronic progressive inflammatory disorder with obligatory damage to the spine and sacroiliac joints [1]. The pathological process often involves entheses, peripheral joints, and other organs and systems. It is necessary to note that the development of magnetic resonance imaging (MRI) diagnostic methods revealed that the disease appears long before radiographic changes become visible. Therefore, according to the most recent nomenclature of spondyloarthritides established in 2023, the term AxSpA unites radiographic axSpA (also known as ankylosing spondylitis) and non-radiographic axSpA [2]. The disease is widespread (20–161 cases per 10,000 population) and affects mostly young people [3].

Alongside inflammation, the pathologic process includes bone tissue remodeling [4]. Inflammatory cytokines disrupt bone tissue formation and resorption processes both locally and systemically [5], depending on the microenvironment and the type of cytokines involved in the inflammatory process [6]. Particularly in axSpA, the inflammatory process in the spine and sacroiliac joints is followed by joint ankyloses and syndesmophytes development. These pathologic features result in limited lability of the spine and peripheral joints and sustained disability in the patients. Otherwise, the disease can be accompanied by systemic osteoporosis and loss of bone mass with subsequent low-energy fractures. An imbalance between bone formation and bone resorption is pivotal for determining the pathway of the AxSpA development [7,8]. The methods of estimation and control of bone tissue remodeling in AxSpA have not yet been developed, and the search and implementation of specific biomarkers remain acute tasks [8].

One of the main regulatory ways in the bone metabolism is a Wnt pathway of osteoblast activation. Serum Wnt protein binds to the membrane LRP 5/6 co-receptor, which destabilizes the protein complex responsible for β-catenine degradation and increases the intracellular amount of the latter. Within the cell nucleus, β-catenine interacts with transcription factors and activates the expression of genes responsible for inhibition of the apoptosis of osteoblasts and stimulation of their growth and differentiation [9]. A soluble serum Dickkopf-1 protein (DKK-1) is a Wnt-signaling antagonist that binds the Wnt receptor and blocks the ligand/receptor interaction, providing β-catenine degradation and turning off the corresponding signaling pathway [10]. DKK-1 is now considered a crucial regulator of bone metabolism in systemic inflammatory disorders [11,12]. While osteogenesis and osteoresorption are guided as well by sclerostin, the bone morphogenic proteins (BMPs) family, and the osteoprotegerin/RANKL/RANK system [13,14,15], DKK-1 has the strongest evidential base. The roles of other bone metabolism markers in the axSpA are less understood. Correlations between DKK-1 serum levels and structural progression in AxSpA or alterations in bone mineral density were established [16,17,18,19]. However, published data obtained with commercial ELISA kits or their home-made analogs currently do not allow one to unambiguously connect serum DKK-1 levels with structural damage in axSpA. The lack of a standard method for DKK-1 level detection worsens the reproducibility of results, and even in healthy volunteers, DKK-1 concentration in serum varies significantly from study to study, in some cases by orders of magnitude [20]. Nevertheless, the utmost importance of this biomarker and necessity of further systemic studies are in no doubt.

Otherwise, nucleic acid aptamers seem to be very promising as recognizing elements of test systems for measuring serum biomarkers. Aptamers are synthetic oligonucleotides with high affinity and selectivity for their specific targets. Their synthetic nature provides batch-to-batch consistency of composition. Moreover, aptamers are relatively stable and well compatible with existing systems for biomarker detection. As a result, aptamer-based test systems represent a viable alternative to existing antibody-based diagnostic kits [21,22]. It should be emphasized that in the field of rheumatology, studying the disease course requires multi-year research with periodic monitoring of certain biomarkers. Antibody-based test systems do not perfectly suit for this purpose due to reproducibility problems that arise from changing the manufacturer of the particular ELISA kit or replacing the antibody component of the kit by its analog with slightly different characteristics [23,24]. From this point of view, test systems that rely on chemically synthesized aptamers with precisely known formulation and highly reproducible characteristics seem to be especially useful for longitudinal studies in rheumatology.

Recently, a single example of a DKK-1-binding DNA aptamer suitable for the creation of a colorimetric test system has been published by Zhou et al. [25]. The authors developed an aptamer/antibody ELISA-like sandwich test system for serum DKK-1 detection in patients with hepatocellular carcinoma.

In the present study, we aimed to widen the toolkit of DKK-1-specific aptamers to obtain novel aptamer-based colorimetric systems for serum DKK-1 detection in patients with ankylosing spondylitis and to test the possibility of their application. To this purpose, we generated new DNA aptamers for DKK-1 with high affinity by in vitro selection from a patterned DNA library and tested the possibility of detecting the target protein by aptamer/antibody and aptamer/aptamer sandwich pairs.

## 2. Results

### 2.1. In Vitro Selection

#### 2.1.1. Library Design

In our study, for the creation of an initial DNA library, we employed a concept of combinatorial library design with purine/pyrimidine alterations proposed by Ruff et al. [26]. In particular, we shortened the randomized region of the combinatorial library from 60 to 40 nucleotides, assuming that such length would provide a proper balance between the diversity and complexity of possible structures and the cost-efficiency of synthesis. Our DNA library included a 40-nucleotide randomized region with a YR pattern (alternating pyrimidines (Y) and purines (R)) interlaced with the stretches of uniform randomization. Moreover, every position in the YR fragments was doped by nucleotides of another type: C + T positions were doped by 5% A and 5% G during the chemical synthesis, and vice versa, purine positions were analogously doped with pyrimidines. This pattern of randomized regions increases the possibility of effective structure formation while maintaining sufficient diversity to generate a large pool of unique sequences.

#### 2.1.2. DNA SELEX

For selection, a recombinant DKK-1 protein with an N-terminal His-Tag was immobilized on Ni-NTA magnetic beads, thus providing magnetic separations during the SELEX cycle. Every selection round included the counter-selection step against the protein-free beads to remove the pool of bead-binding molecules. To suppress non-specific biomolecular interactions, we supplied the selection buffer with human serum albumin (HSA) and polyA.

**Figure 1:**
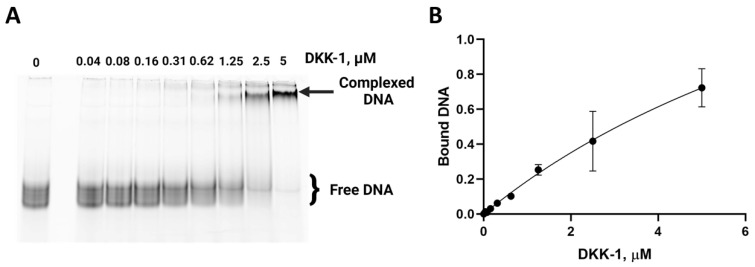
An assessment of library enrichment evaluation after the 4th round of selection: Non-denaturing PAGE analysis of Cy5-labeled DNA–protein complexes (**A**) and corresponding binding curve generated with a bimolecular binding equation in GraphPad Prism 8.0.1 software (**B**).

After the fourth SELEX round, we assessed the efficacy of the selection process. Electrophoretic mobility shift assay (EMSA) was performed to analyze the formation of complexes with DKK-1 using the fluorescently labeled DNA (Figure 1) generated with the use of a corresponding primer bearing a cyanine dye. As protein concentration increased, we observed the accumulation of products with low electrophoretic mobility, which correspond to DNA–protein complexes. We concluded that the enriched DNA pool after the fourth round contains a significant fraction of molecules with affinity for the target. Therefore, it was subjected to middle-throughput sequencing on the MiSeq Illumina platform to validate the enrichment and identify individual aptamer candidates.

The analysis of the obtained data showed an apparent accumulation of specific DNA sequences. The top twenty most represented sequences of the central randomized region are given in Table 1. In most positions, selection leaders preserved the pre-defined pattern of the randomized region with purine–pyrimidine alterations. However, doping purine sites with pyrimidine nucleotides and vice versa prove to be functionally advantageous. The median number of transversions (replacements of dominating R in a certain position by doping Y or dominating R by Y) per sequence was found to be 3.5.

**Figure 2:**
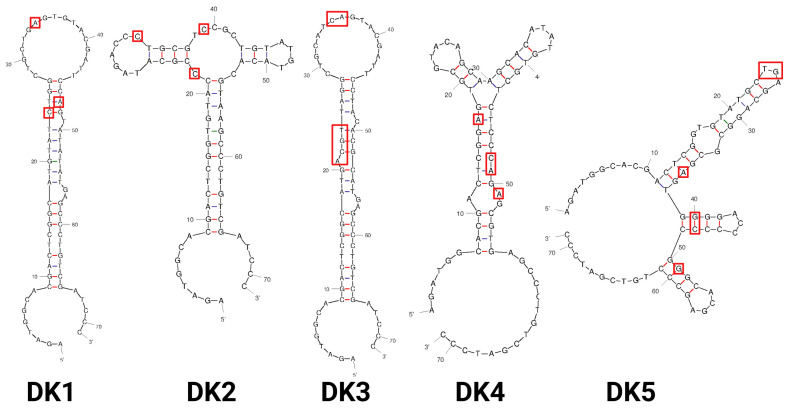
The most probable secondary structures of aptamers DK1–DK5 were obtained with the mfold web server [27]. Transversions in the pyrimidine–purine alternation pattern are shown in red boxes.

After an analysis of secondary structures generated with the mfold web server [27], we identified two distinctive groups (see Figure 2 for aptamers DK1–DK5 and Figure S1 for DK6–DK20). The first group comprises more than a half of the top twenty aptamers: DK1, DK3, DK7, DK8, DK10, DK11, DK13-DK17, DK19, and DK20. Their structures contain a common scaffold comprising a long stem-loop with several 1–2 nt bulges and internal loops. It should be noted that, alongside similar secondary structures, aptamers DK1 and DK3 also possess significant sequence similarity. The second group is smaller and includes the aptamers DK2, DK4, DK6, DK9, and DK18, sharing the common structural element of a three-way junction built by three stem-loops. The most extended stem contains several small internal loops. On the contrary, aptamers DK5 and DK12 did not fall into any of these groups.

### 2.2. Aptamer Screening

We took the five most represented sequences, DK1–DK5, for chemical synthesis and affinity screening (Figure 2). To provide the possibility of further terminal modifications, the candidate aptamers were supplied by a 3′-aliphatic amino linker.

**Figure 3:**
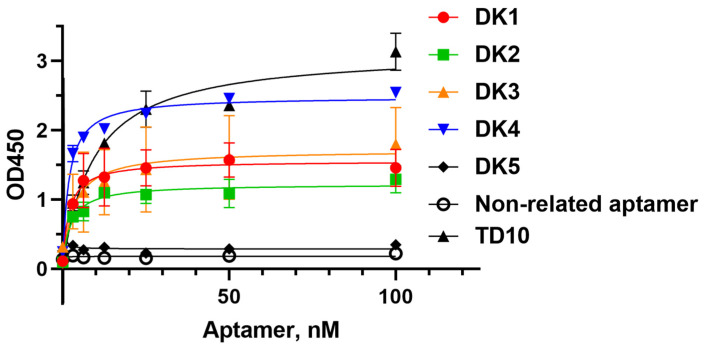
ELASA analysis of aptamer binding with DKK1. Microplate wells were coated with 2 μg/mL DKK-1; the concentration of aptamers varied from 3.12 nM to 100 nM. Each data point represents the average value of two independent experiments.

We employed an ELASA (enzyme-linked aptamer sorbent assay) to assess the affinity of candidate aptamers. To confirm the binding specificity of candidates, we used a non-related DNA aptamer against the IL-17A receptor [28]. For a comparison, we also evaluated in the same assay the affinity of TD10, a previously published aptamer against the DKK-1 [25]. Although the data from [25] suggest that this aptamer has a rather high affinity for DKK-1, the authors have not provided any quantitative characteristics. Binding curves are given in Figure 3, and the values of corresponding dissociation constants are listed in Table 2. Aptamers DK1, DK2, DK3, and DK4 showed high affinity for DKK-1 with K_D_ values in the low nM range. Within all concentration ranges, we did not register a signal growth for DK5 and control aptamers (Figure 3), which confirms the specificity of aptamer–protein binding. In the wells coated only by bovine serum albumin (BSA), we registered only a background signal, which also confirms binding specificity. Aptamer TD10 also demonstrated a high affinity for DKK-1, comparable to that of our newly generated aptamers.

### 2.3. Aptamers’ Truncation

**Figure 4:**
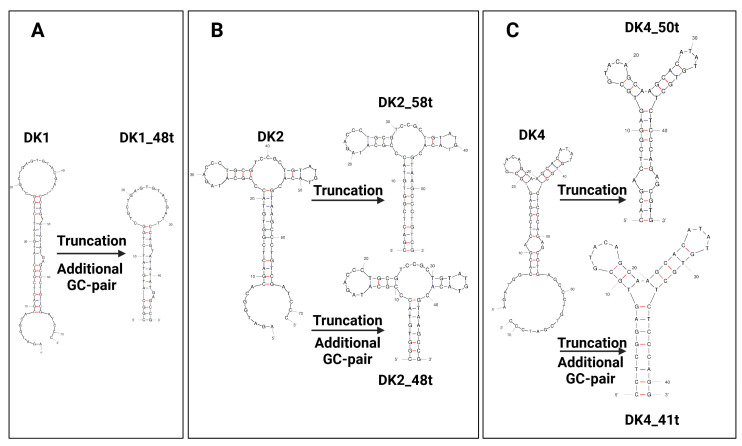
Schematic representation of rational truncation for aptamers DK1 (**A**), DK2 (**B**), and DK4 (**C**). AT pairs are shown by black lines, GC pairs by red lines.

The truncation of aptamer sequences makes their chemical synthesis faster and more economic. It also reduces the probability of the formation of non-productive secondary structures. As a rule, rational sequence design becomes the next step after the primary assessment of candidate aptamers after selection. Nevertheless, there are no universal algorithms for optimization and modification of the aptamer nucleotide sequence after selection. The task of revealing structure elements essential for target binding requires a specific strategy for every aptamer [29]. Commonly used approaches to the post-SELEX design include combining scaffolds from different aptamers to improve binding affinity or consecutive elimination of terminal nucleotides to identify a minimal structure sufficient for target binding [30,31]. In this study, we truncated aptamer sequences, aiming to: (1) preserve the central scaffold (stem-loop or three-way junction); (2) remove as much as possible single-stranded fragments; and (3) shorten double-stranded ones. The schemes of truncation are shown in Figure 4. In some cases, we supplied the stem with an extra closing GC-pair that stabilized the secondary structure. Using this strategy, we generated a series of truncated variants for the high-affinity aptamers, DK1, DK2, and DK4 (Table 3). Truncated aptamers were chemically synthesized as 3′-NH_2_-derivatives and then biotinylated.

**Figure 5:**
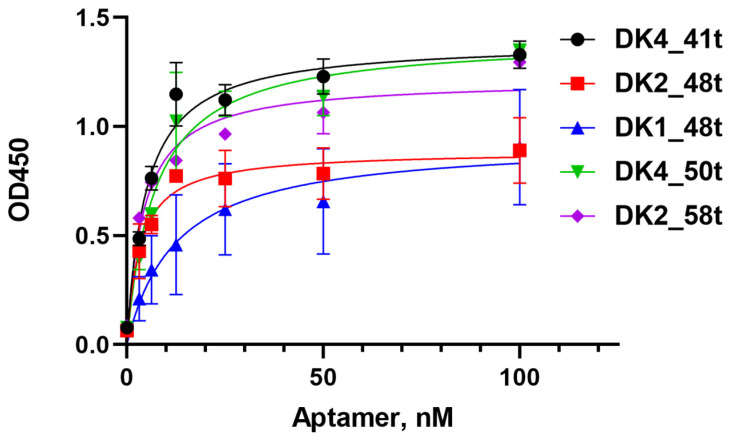
ELASA analysis of truncated DNA aptamer binding with DKK1. Microplate’s wells were coated with 2 μg/mL DKK-1; the concentration of aptamers varied from 3.12 nM to 100 nM. Each data point represents the average value of two independent experiments.

To evaluate their affinity, we used the ELASA method described above with protein–target immobilization on the plate surface. As we can see from K_d_ values, all five truncated aptamers, DK1_48t, DK2_48t, DK2_58t, DK4_50t, and DK4_41t, kept high affinity for DKK-1 (Figure 5), with only a small rise of the dissociation constants as compared to their parent aptamers (Table 2).

### 2.4. Development of Colorimetric Sandwich System for DKK-1 Detection

**Figure 6:**
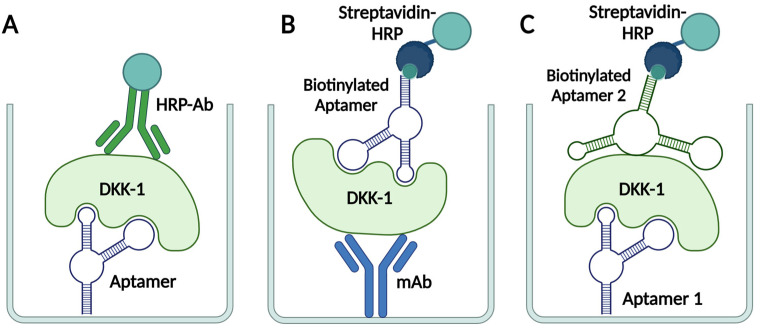
Schematic representation of colorimetric sandwich systems for DKK-1 detection, based on aptamer/antibody (**A**), antibody/aptamer (**B**), or aptamer/aptamer (**C**) pairs.

We tested several of the newly selected aptamers (aptamers DK1, DK4, and truncated aptamers DK1_48t and DK4_41t) as biospecific units in two types of ELISA-like sandwich systems for DKK-1 detection (Figure 6), with another aptamer or antibody as a second specific component.

**Figure 7:**
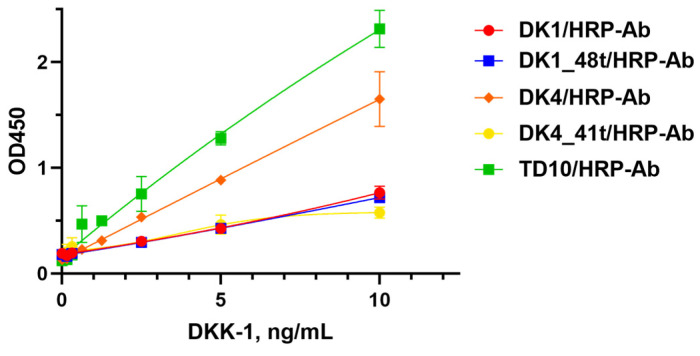
Dependencies of optical density from DKK-1 concentration for aptamer/antibody sandwich systems; the GraphPad Prism 8.0.1 software package was used to plot the 4PL curve. Each point represents the average value of two independent experiments.

In the first case, one of the aptamers was covalently immobilized on the carboxy-modified plate surface via the terminal amino linker as a capture component. Polyclonal anti-DKK-1 antibodies conjugated with horseradish peroxidase (HRP) served as a reporter component (Figure 6A). For all tested aptamers, we obtained a clear and prominent growth in color intensity with an increase in DKK-1 concentration in the range of 0.156–10 ng/mL (Figure 7), which corresponds to the working range of commercial DKK-1 ELISA kits. Under the same conditions, an analogous assay with TD10 aptamer also demonstrated concentration-dependent signal growth. The DK4/antibody and TD10/antibody combinations provided the highest signal magnitude and sensitivity among all aptamer/antibody variants. We also proved the assay specificity by replacing the DKK-1 with another soluble serum protein, interleukin-17A (Figure S2). Only a faint signal, not higher than background values, was registered up to 200 ng/mL.

On the contrary, after turning the system upside down with a DKK-1-specific antibody non-covalently immobilized on a plate surface and a biotinylated aptamer for recruiting HRP–streptavidin conjugate (Figure 6B), we did not register an analytical signal. We hypothesize that surface-bound antibodies could lose their ability for DKK-1 binding.

In order to take full advantage of the benefits of aptamers in DKK-1 detection, we have developed a colorimetric test system variant based on aptamer pair combinations, using DK1, DK4, DK1_50t, DK4_41t, and TD10 aptamers. The first aptamer was covalently immobilized on the plate surface as described above, and the second biotinylated aptamer acted as a reporter in combination with streptavidin–HRP conjugate (Figure 6B).

For each of the six aptamer pairs, we examined both possible variants of the sandwich. A growth of optical density with the increase of protein concentration showed the workability of the aptamer-only assays. The replacement of DKK-1 by another protein, interleukin-17A, gave very low values of the optical density (Figure S2) and thus proved the assay specificity.

**Figure 8:**
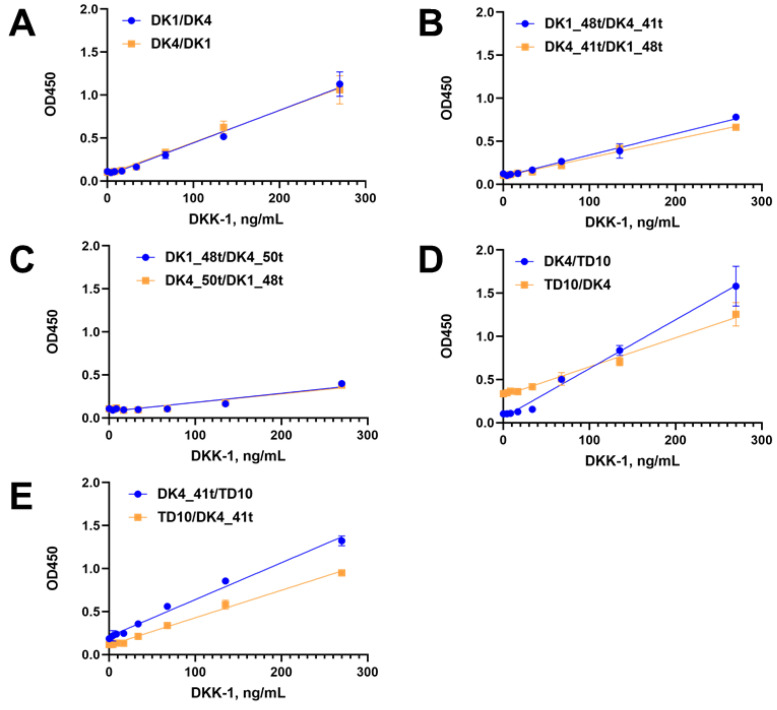
Dependencies of optical density from DKK-1 concentration for aptamer/aptamer sandwich pairs DK1/DK4 (**A**), DK1_48t/DK4_41t (**B**), DK1_48t/DK4_50t (**C**), DK4/TD10 (**D**), and DK4_41t/TD10 (**E**). The GraphPad Prism 8.0.1 software package was used to plot the linear dependency of OD450 from DKK-1 concentration. Each point represents the average value of two independent experiments.

Interestingly, for the TD10/DK4-based system, we observed a rather high background signal in the absence of the protein (Figure 8D). Low signal magnitudes were registered for combinations of the DK1 and DK4 truncated variants (Figure 8B,C). The system comprising surface-immobilized DK4_41t and reporter TD10 (Figure 8E) provided the best linear response with a high magnitude of optical density. However, the concentration range of DKK-1 was 0.156–10 nM, which corresponds to 4.2–270 ng/mL. A comparison of this concentration range with the one for the aptamer/antibody sandwich showed that the aptamer/aptamer system is less sensitive and needs additional optimization to be used for the analysis of clinical samples.

### 2.5. Serum Samples Analysis

**Figure 9:**
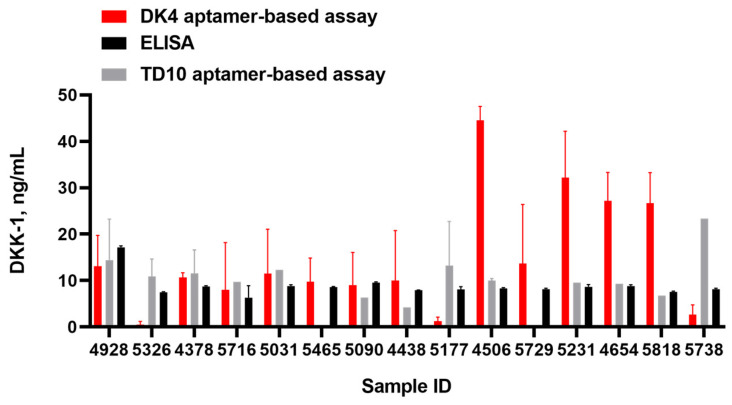
Results of the DK4-based (red bars), TD10-based (gray bars), and ELISA (black bars) analyses of the DKK-1 levels in blood serums diluted by PBS (1:10). The values of DKK-1 concentration in the samples were calculated with the use of the online service https://www.myassays.com/four-parameter-logistic-curve.assay (accessed on 7 November 2024). Each point represents the average value of two independent experiments.

Finally, we examined the possibility of using the aptamer/antibody colorimetric assays DK4/HRP-Ab and TD10/HRP-Ab in the analysis of blood serum from AxSpA patients. As a reference, we used a commercial ELISA kit for serum DKK-1 detection. Each blood serum sample was diluted 10-fold by the PBS buffer and used either for an aptamer/antibody assay or for ELISA without further preprocessing. The aptamer/antibody assays were performed according to the protocol described above (Section 2.4), using the calibration curves analogous to those presented in Figure 7. The results are shown in Figure 9.

The values of a DKK-1 level are in most cases within the same order of magnitude and show quite a good coincidence between two aptamer-based assays and reference ELISA. The obtained values fall into the typical range for DKK-1 serum concentration. However, in a number of cases we registered significant differences between assays based on TD10 and DK4 aptamers. Namely, the samples 5326, 5177, 4506, 5729, 5231, 4654, and 5818 gave higher values for the DK4-based assay, while the sample 5738 showed a higher DKK-1 level in a TD10-based system.

## 3. Discussion

The selection of aptamers capable of forming sandwich pairs for the detection of protein targets is a complex task and can require several selection procedures. Currently, the examples of aptamer/aptamer sandwich assays are still few in number. In particular, colorimetric or electrochemical aptamer-only sandwich assays were reported for thrombin, platelet-derived growth factor, and vaspin [32]. U. Ochner et al. [33] generated sandwich pairs of slow off-rate modified aptamers (SOMAmers) for the biomarkers of cardiovascular risk and employed them for target detection on a Luminex platform. A pair of troponin I-binding DNA aptamers, tro4 and tro6, became the basis for electrochemical and surface-enhanced resonance Raman scattering assays [34]. Alternatively, two spiegelmers to the troponin I were used in an AlphaLisa fluorescent assay [35].

Every SELEX procedure starts with a choice of combinatorial DNA or RNA library. Up to now, a smooth, uniform randomization of the core remains the most popular approach in the design of libraries for in vitro selection. Nevertheless, the use of partly structured libraries or those with preformed secondary structure elements gives a possibility to improve selection efficiency, reduce the number of selection rounds, and obtain high-affinity binders [36]. Here, we adapted the combinatorial library design with purine/pyrimidine alteration motifs in the randomized region, proposed by Ruff et al. [26]. This strategy showed quite high efficiency, since in only four rounds of selection we were able to obtain a noticeable enrichment of the library by DKK-1-binding DNAs. Our results coincide well with those obtained by Ruff et al. [26] and prove the importance of doping of all pattern positions by other nucleotide types. A doping of predominant nucleotides in alternating positions by a small fraction of nucleotides of another type (e.g., purines doped by pyrimidines) allows each specific aptamer to acquire a transversion during the sequence evolution, if it is preferable for a better binding. After analyzing the most probable secondary structures of selection leaders (Figure 2 and Figure S1), we did not find any preferable sites for transversions. They are located in both single-stranded and double-stranded fragments. We hypothesize that deviations from the general alternation pattern provide additional stabilization of certain structural elements by lengthening double-stranded regions. On the other hand, the transversions may locally improve the flexibility of the aptamer via increasing the length of loops or introducing bulges. It could, in turn, promote the fine tuning of shape complementarity and enhance binding efficiency.

We did not pinpoint clear structural similarities between the selection leaders and the previously described DKK-1-specific aptamer TD10 [25]. However, the sequences of DK11, DK14, and DK15 contain a consensus binding motif, TGTAA, identified by Zhou et al. [25]. Further secondary structure study revealed that the TGTAA motif is accessible for binding and does not contribute to the formation of double-stranded secondary structure elements for DK14 and DK15. As such, they could also be promising binders; however, more research is required to determine their affinity for DKK-1.

Among the top five most represented aptamers, four (DK1, DK2, DK3, and DK4) possessed prominent DKK-1 binding affinity with low nanomolar K_D_ values and binding specificity, as confirmed by an absence of binding with another soluble protein, interleukin-17A. As a next step, we performed a post-SELEX rational sequence design to remove structural elements that are not crucial for target binding (Figure 4). The DKK-1 binding assay showed that the DK1 aptamer is the most sensitive for truncation; a noticeable decrease in affinity was found for its shortened version, DK1_48t (Kd 1.3 nM and 10 nM, respectively). It can be assumed that the contribution to binding to DKK-1 comes not only from the structure with the lowest energy, which was used for the rational design of the truncated aptamer, but also from alternative structures that were lost when the length was shortened. Otherwise, the aptamers DK2 and DK4 were successfully shortened from 71 nt to 48 nt or 41 nt, respectively, with only a minor loss of binding affinity.

We employed the generated novel DNA aptamers together with the aptamer TD10 obtained by Zhou et al. [25] to create a series of ELISA-like colorimetric assays. Aptamer/antibody test systems demonstrated rather high sensitivity. The use of DK4 and TD10 aptamers as capture elements immobilized on a plate surface allowed for DKK-1 detection in the concentration range 0.156–10 ng/mL, comparable to that of commercially available ELISA kits for DKK-1 detection. We also tested the possibility of employing two aptamers as both capture and reporter components of the sandwich ELISA-like assay to make it antibody-free. Indeed, several aptamer/aptamer pairs, namely DK4/TD10, DK4_41t/TD10, and DK1/DK4 pairs, provided a linear growth of the colorimetric signal with the increase in a DKK-1 concentration. Thus, we might propose that these aptamers bind different epitopes of the DKK-1 protein. However, the sensitivity of these aptamer/aptamer colorimetric assays is still inferior to that of aptamer/antibody analogs and needs further optimization.

We examined the aptamer/antibody sandwich systems in the analysis of real clinical samples, specifically a series of blood serums from patients with a confirmed diagnosis of AxSpA, to assess their potential for further applications. The reference commercial ELISA kit, used as an independent reference, gave DKK-1 concentration values in the range of 6–18 ng/mL. These results are comparable with a mean value of DKK-1 level in axSpA patients (5.4 ng/mL) measured by the same method in the DESIR multicenter cohort study [37]. Both aptamer-based assays provided the DKK-1 level values consistent with those obtained by ELISA. However, we also registered a number of cases where two aptamer-based assays gave different values of the DKK-1 level for the same serum sample. We assume that this effect might point to the modest sensitivity of aptamer-based assays at low DKK-1 concentrations. Meanwhile, both TD10 and DK4 aptamers were selected with the use of recombinant protein as a target. We can hypothesize that the aptamers recognize the glycosylated protein in the human blood with different affinities as compared to the recombinant DKK-1. This hypothesis surely needs proving and will be the subject of our further studies. It is also worth noting that conventional reference values of DKK-1 serum level in healthy people and axSpA are still not established, owing also to a lack of the standard assay. We assume that the development of aptamer-based DKK-1 detection assays would contribute to solving this problem.

## 4. Materials and Methods

### 4.1. Materials and Reagents

Recombinant active human DKK-1 protein, monoclonal anti-DKK-1 antibodies, polyclonal anti-DKK-1 antibodies conjugated with HRP, and an ELISA kit for human DKK-1 were purchased from Cloud-Clone Corp. (Wuhan, China). His Mag Sepharose Ni magnetic beads were purchased from GE Healthcare (Chicago, IL, USA). 5′,N-protected 2′-deoxyribophoshporamidites, 3′-PT-Amino-Modifier C6, and Spacer Phosphoramidite 18 were purchased from Glen Research Inc. (Sterling, VA, USA). Phosphate-Buffered Saline (PBS) tablets were purchased from VWR (Radnor, PA, USA). 1-Methylimidazole, N-(3-dimethylaminopropyl)-N′-ethyl carbodiimide hydrochloride, Tween 20, HSA, and biotin N-hydroxysuccinimide (NHS) ester were purchased from Sigma-Aldrich (St. Louis, MO, USA). 3,3′,5,5′-Tetramethylbenzidine (TMB) was purchased from Fluka (Waltham, MA, USA). The conjugate of streptavidin with HRP was purchased from IMTEK (Moscow, Russia). Cy5 NHS ester was purchased from Lumiprobe (Moscow, Russia). HotStart Taq DNA polymerase, deoxyribonucleoside triphosphates, and 10x PCR buffer were purchased from Biolabmix (Novosibirsk, Russia).

High-binding clear 96-well plates for protein immobilization were purchased from Greiner (Kremsmünster, Austria). Nunc™ NucleoLink™ microplate strips for oligonucleotide immobilization were purchased from Thermo Fisher Scientific (Waltham, MA, USA).

### 4.2. Synthesis of Oligonucleotides

DNA aptamers, primers, and combinatorial DNA libraries were synthesized by the solid-phase phosphoramidite method on a 0.4 µmol scale on an automated DNA/RNA synthesizer ASM-800 (Biosset, Novosibirsk, Russia) using corresponding 5′,N-protected phosphoramidites of deoxyribonucleotides and optimized protocols. During the synthesis of the combinatorial region of the initial library, we used phosphoramidite mixtures with A:C:G:T proportions depending on the position (see Section 4.3). Oligonucleotides bearing the 3′-amino group were synthesized using the modified polymer support 3′-PT-Amino-Modifier C6 CPG and Spacer Phosphoramidite 18. After synthesis, oligonucleotide separation from polymer support and deprotection were carried out with 300 µL of 40% aq. methylamine solution at 65 °C for 15 min. Completely deprotected DNA aptamers, primers, and the initial library were purified by denaturing PAGE.

### 4.3. In Vitro Selection of DNA Aptamers

In vitro selection was carried out using the combinatorial ssDNA library 5′-AGATGGCACGACTCGG-(YR)_5_-N_5_-(YR)_5_-N_5_-(YR)_5_-AGCCCTGTCGATCCC-3′, with the following A:C:G:T proportions in the randomized region: N—1:1:1:1, Y—5:45:5:45, R—45:5:45:5. For each round of selection, 1 nmol of DNA library was folded in 1× PBS, pH 7.4, 5 mM MgCl_2_ by heating to 95 °C for 5 min, followed by snap-cooling on ice for 5 min and incubation at 25 °C for 15 min, then supplemented with 0.05% Tween 20, 100 μg/mL polyA, and 0.01% HSA (total volume 200 μL) and incubated at 25 °C for an additional 15 min. Moreover, 2 μg of DKK-1 were immobilized on HisMag Sepharose Ni magnetic beads (15 μL of 5% slurry). Every round of SELEX included a negative selection step: a folded DNA library was incubated with magnetic beads without protein for 30 min at room temperature with mixing. An unbound DNA pool was separated from beads using magnetic separation, collected, and incubated with DKK-1-loaded magnetic beads for 1 h at room temperature with mixing. The unbound DNA pool was isolated using magnetic separation and discarded. Beads with DNA/DKK-1 complexes were washed 3 times with 200 μL of PBSMT (1× PBS, pH 7.4, 5 mM MgCl_2_, 0.05% Tween 20). To elute complexes, 15 μL of 20 mM Tris-HCl, pH 7.5, and 100 mM imidazole were added to the beads and incubated for 15 min at room temperature with mixing. After magnetic separation, supernatant was collected, supplemented with PCR buffer, 7.5 mM MgCl_2_, 1 mM dNTPs, 1 μM each forward (5′-AGATGGCACGACTCG G-3′) and reverse (5′-GGGATCGACAGG GCT-3′) primers, 2.5 U HotStart Taq DNA polymerase, and amplified: 94 °C—3 min, 94 °C —30 s, 50 °C—30 s, 72 °C—30 s (10 cycles), 72 °C—3 min. Asymmetric PCR (aPCR) with only a forward primer was used to generate an enriched ssDNA library, followed by purification using Amicon Ultra-0.5 10 K (Merk Millipore, Burlington, MA, USA). A total of 4 rounds were performed. Detailed conditions for each round are given in Table S1.

### 4.4. Assessment of Enriched Library Affinity by Non-Denaturing Gel Electrophoresis

The 5′-Cy5-labeled enriched ssDNA library was generated via asymmetric PCR with a 5′-Cy5-bearing forward primer. Fluorescent dye was incorporated at the 5′-terminus of the primer using Cy5 NHS ester by optimized manufacturer’s protocol.

The Cy5-labeled library (0.2 μM) was heat-folded in binding buffer (1× PBS, pH 7.4, 10 mM MgCl_2_, 0.1% Tween 20, 200 μg/mL polyA). Moreover, 5 μL of 0–10 μM DKK-1 solution were added to 5 μL of the folded Cy5-labeled library (final concentration 0.1 μM) and incubated for 1 h at room temperature. Then, 2.5 μL of loading buffer (0.05% bromophenol blue in 30% glycerol) was added to the samples, followed by loading on 12% native PAGE (49:1, 0.5x TB buffer). The results of electrophoresis were visualized with the Amersham Typhoon scanner (GE Healthcare, Chicago, IL, USA). Images were processed with the Quantity One 4.5.1 software package (Bio-Rad, Hercules, CA, USA). A bimolecular binding equation in the GraphPad Prism 8.0.1 software package (GraphPad Software, Boston, MA, USA) was used to plot the binding curve.

### 4.5. Illumina High-Throughput Sequencing and Data Analysis

Enriched dsDNA libraries (90 ng) were ligated with adapters using the NEBNext Ultra II DNA Library Prep Kit for Illumina and barcoded by NEBNext Multiplex Oligos (NEB). The obtained libraries were sequenced on a MiSeq platform with MiSeq Reagent Kit v3 (600-cycle) (Illumina) in the SB RAS Genomics Core Facility (ICBFM SB RAS, Novosibirsk, Russia) with coverage > 150,000 paired reads/sample. Raw read sequences were analyzed with the UPARSE pipeline [38] using Usearch v11.0.6670.0.240. The UPARSE pipeline included the merging of paired reads; read quality filtering; length trimming; merging of identical reads (dereplication); discarding singleton reads; removing chimeras; and OTU clustering.

### 4.6. Enzyme-Linked Aptamer Sorbent Assay (ELASA)

For enzyme-linked assays, biotinylated aptamers were synthesized using biotin NHS ester, as described in [39].

100 μL of 2 μg/mL DKK-1 solution in 1× PBS, pH 7.4, were immobilized in the wells of the high-binding plate at 4 °C overnight. The plate was washed 3 times with 200 μL of PBSMT, then the surface was blocked with 150 μL of 1% bovine serum albumin (BSA) in 1× PBS for 2 h at 25 °C, followed by a wash. Furthermore, 100 μL of refolded biotinylated aptamer in PBSMT were added and incubated for 1 h at 25 °C. After washing, 100 μL of 0.2 μg/mL streptavidin–HRP solution in 1× PBS was added to the wells. The plate was incubated for 1 h at 25 °C, washed, and then 100 μL of 0.2 mg/mL TMB solution in 0.1 M sodium acetate with 0.03% H_2_O_2_ were added and incubated for 20 min at 25 °C. Moreover, 100 μL of 10% H_2_SO_4_ were added for color fixation, followed by the measuring of the absorbance at 450 nm by a microplate reader AMR-100 (AllSheng, Hangzhou, China).

A bimolecular binding equation in the GraphPad Prism 8.0.1 software package was used to plot binding curves and calculate dissociation constants.

### 4.7. Sandwich Aptamer-Based Assay for DKK-1 Detection

3′-Amino modified aptamers were immobilized in NucleoLink™ wells in the following way: 100 μL of a freshly made solution containing 250 nM aptamer, 10 mM 1-methylimidazole, and 10 mM EDC were incubated for 16 h at 50 °C. Then, wells were washed three times with 300 μL of PBSMT.

After immobilization, the wells were blocked with 250 μL of 0.5% PEG-8000 in PBSMT for 1.5 h at 25 °C and washed 3 times. Moreover, 100 μL of 0–10 nM DKK-1 in PBSMT were added and incubated for 1 h at 25 °C, then the solution was removed.

A biotinylated aptamer or polyclonal anti-DKK-1 antibody conjugated with HRP was used as a secondary component of the sandwich pair.

In the first case, 100 μL of 200 nM folded biotinylated aptamer in PBSMT were added and incubated for 1 h at 25 °C. Wells were washed three times, and 100 μL of 0.4 μg/mL streptavidin–HRP in PBSMT were incubated for 1 h at 25 °C. Then, the solution was discarded, and wells were washed three times.

In the second case, 100 μL of 1:500 diluted antibody–HRP conjugate in PBSMT were incubated for 1 h at 25 °C. Then, the solution was discarded, and wells were washed three times.

To generate a colorimetric signal, TMB solution was used as described above (Section 4.6), followed by measuring the absorbance at 450 nm.

### 4.8. DKK-1 Detection in Serum Samples

Blood collection and all instrumental investigations were performed after the signing of informed voluntary consent. This study was conducted in accordance with ethical standards developed in accordance with the Helsinki Declaration of the World Medical Association, “Ethical Principles of Scientific Medical Research with Human Participation”, as amended in 2013, and “Rules of Clinical Practice in the Russian Federation”, approved by the Order of the Ministry of Health of the Russian Federation from 01.04.2016 № 200n, and also approved by the Local Ethics Committee of the Research Institute of Clinical and Experimental Lymphology. This study included 18 patients with AS at an early or advanced stage of the disease. Blood was collected into the standard 5 mL vacutainers with separation gel and coagulation activator. Samples were centrifuged for 10 min at 3000 rpm; the resulting serums were split into 500 μL aliquots and stored at −20 °C.

The microplate colorimetric analysis of serum samples with DK4 aptamer/antibody sandwiches was performed according to the protocol described above in Section 4.7. Prior to analysis, the samples were diluted 1:10 times with 1× PBS. Two parallel measurements were made for each sample. As a reference, we used a commercially available ELISA kit for human DKK-1 according to the manufacturer’s instructions. In both cases, we applied the four-parameter logistic curve (4PL) equation from the Web service https://www.myassays.com/four-parameter-logistic-curve.assay accessed on 7 November 2022) to plot calibration curves and determine DKK-1 concentration in serum samples.

## 5. Conclusions

Our investigation confirmed that employing a DNA library with purine/pyrimidine alternations in the randomized region enhances selection efficiency and reduces the number of SELEX cycles. We generated a series of high-affinity DNA aptamers for DKK-1 and developed their truncated variants that retained binding affinity. It was shown that novel DNA aptamers can act as capture components in a microplate ELISA-like assay with HRP-conjugated anti-DKK-1 antibody as a reporter component. Moreover, we succeeded in forming the aptamer/aptamer sandwich pairs that provided an aptamer-only sandwich colorimetric assay, although the sensitivity of the assay still requires improvement.

For the aptamer/antibody assay format, we also examined the test systems based on DK4 and previously published TD10 aptamers for analyses of blood serum from AxSpA patients. Both aptamers provided the results that fall into the typical range for DKK-1 serum concentration and are comparable to those obtained by a reference ELISA kit. However, in a number of cases, we registered significant differences between assays based on TD10 and DK4 aptamers. We hypothesize that this effect might originate from the modest sensitivity of aptamer-based assays at low DKK-1 concentrations or with different abilities of the aptamers selected against the recombinant DKK-1 to recognize the glycosylated protein in the human blood.

Nevertheless, the obtained aptamers to the DKK-1 protein showed good potential as components of test systems for determining the DKK-1 level in blood serum. Future studies in the field have to be directed to clarifying the problem of different results obtained for model recombinant proteins and real molecules in blood and to improving the sensitivity of aptamer-based assays in a manner that remains compatible with clinical diagnostics.

## 6. Patents

Nucleotide sequences of the aptamers DK1, DK2, DK4, DK4-41t, and DK2-48t are the subject of RU patent # 2814580, priority date 28 July 2023.

## Figures and Tables

**Table 1:** The top twenty most represented sequences corresponding to the central randomized region.

Initial Pattern	YRYRYRYRYRNNNNNYRYRYRYRYRNNNNNYRYRYRYRYR	
ID	Randomized Region Sequence 5′->3′	% *
DK1	CATGTAT**C**TGGCTGCTG**A**GTGTACGATTCC**A**GTATATATG	2.29
DK2	TGTAC**C**CGCATAGACC**C**TGCGT**C**CGCTGTATGTACACGTA	2.17
DK3	CATG**ACGT**TAGGCTGCAT**CA**GTACGATTCCTACACGCATG	1.84
DK4	**A**GTGCGTACAGCAAGCACATATGTGCTCTCC**CA**G**A**GCGTG	1.52
DK5	TGTATGC**T**GAGCAGGCGCG**A**GTG**G**GGACCCC**C**CG**G**GCACG	1.36
DK6	CGTATG**A**ACGGGGACC**C**TATG**G**GCGTTTGCTATGCACATA	1.24
DK7	CGTGCATACGAGCCGCATGTACACATTCTCTGTATGTACG	1.04
DK8	CACGTGCG**A**AGTGGTTA**AC**CATG**GC**CTAGTC**C**CGCA**A**GTG	1.01
DK9	CA**G**G**G**ACACGGGGACC**C**TACG**G**GCGTGTCTCATATACATG	1.00
DK10	CATATATG**A**GAGCCGCGTA**AT**TATACTCTCTGCATGTATG	0.97
DK11	CGTGCATGTAAGCCGCGTATACATACTCTTCAC**T**TGCACG	0.95
DK12	CGTG**G**ATATAGCCGC**A**ACGT**C**CATAGTTTCTA**G**ATACACG	0.94
DK13	CACATATGCAGCCGCTGTGTATGCACATCT**G**ACATGTGTG	0.88
DK14	CG**G**GTG**G**ATACCAGCTGCGTGTA**A**ACTCTGTGTAC**C**C**C**CG	0.82
DK15	CATG**A**GTGTGGTTGCCGCATGTA**A**ATTCAAC**C**CAT**C**CATG	0.82
DK16	CACGTGTATGAAAGCCGCGTATATACTCTTTAC**T**T**C**CGTG	0.80
DK17	CATA**G**GTATACGCGCTGCGT**C**TACGCAACGTACAC**T**CATG	0.74
DK18	TAC**C**TG**A**GCAGGGGTTG**G**ACG**G**G**G**ACCCTCTG**G**GCGTAC**T**	0.71
DK19	CACATACGT**C**AGCCGCATGTGTATACATTCTGTGT**T**TGTG	0.70
DK20	CATATGT**C**TGCCGGG**GT**C**C**C**T**TATGGGCGGCACGT**C**TATG	0.67

**Bold underlined symbols (N)**—purine–pyrimidine or pyrimidine–purine transversions, as compared to the initial pattern of the DNA library given at the top of the table. * The frequency of occurrence is calculated as the ratio of the number of DNA copies of this particular sequence to the total number of DNA molecules in the sequenced pool.

**Table 2:** Calculated values of dissociation constants.

ID	K_d,_ nM
DK1	1.3 ± 1
DK2	1.8 ± 1
DK3	3.7 ± 3
DK4	1.8 ± 0.5
DK1_48t	10 ± 6
DK2_58t	3.6 ± 2
DK2_48t	3.3 ± 2
DK4_50t	6.1 ± 2
DK4_41t	5.6 ± 3
TD10	7 ± 5

**Table 3:** Sequences of truncated aptamers.

ID	Truncated Sequence
DK1_48t	CGGCATGTATCTGGCTGCTGAGTGTACGATTCCAGTATATATGAGCCG
DK2_58t	CGACTCGGTGTACCCGCATAGACCCTGCGTCCGCTGTATGTACACGTAAGCCCTGTCG
DK2_48t	CGGTGTACCCGCATAGACCCTGCGTCCGCTGTATGTACACGTAAGCCG
DK4_50t	CACGACTCGGAGTGCGTACAGCAAGCACATATGTGCTCTCCCAGAGCGTG
DK4_41t	CCTCGGAGTGCGTACAGCAAGCACATATGTGCTCTCCCAGG

## Data Availability

The original contributions presented in the study are included in the article/Supplementary Material, further inquiries can be directed to the corresponding author.

## Supplementary Materials

The following supporting information can be downloaded at: https://www.mdpi.com/article/10.3390/ijms252212214/s1.

## Abbreviations

aPCR	asymmetric polymerase chain reaction
AxSpA	axial spondyloarthritis
BSA	bovine serum albumin
CPG	controled pore glass
DKK-1	Dickkopf-1 protein
EDC	N-(3-dimethylaminopropyl)-N′-ethylcarbodiimide hydrochloride
ELASA	enzyme-linked aptamer sorbent assay
ELONA	enzyme-linked oligonucleotide assay
EMSA	electrophoretic mobility shift assay
HRP	horseradish peroxidase
HSA	human serum albumin
NHS	N-hydroxysuccinimide
PBS	Phosphate-Buffered Saline
PEG	poly(ethylene glycol)
SELEX	Systematic Evolution of Ligands by Exponential Enrichment
TMB	3,3′,5,5′-tetramethylbenzidine
4PL	four-parameter logistic regression
