# Supplementary material for: Novel DNA Aptamers to Dickkopf-1 Protein and Their Application in Colorimetric Sandwich Assays for Target Detection in Patients with Axial Spondyloarthritis
Source: Int J Mol Sci. 2024 Nov 14;25(22):12214. doi: 10.3390/ijms252212214 (PMC11594316; doi:10.3390/ijms252212214)
Supplement: Supplementary file 1 [file ijms-25-12214-s001.zip › ijms-3261279-supplementary.pdf]

International Journal of
*Molecular Sciences*

MDPI

*Article*

# Novel DNA Aptamers to Dickkopf-1 Protein and Their Application in Colorimetric Sandwich Assays for Target Detection in Patients with Axial Spondyloarthritis

**Elizaveta A. Shatunova [1], Anastasia S. Rychkova [1], Mariya I. Meschaninova [1], Marsel R. Kabilov [1], Alexey E. Tupikin [1], Yulia D. Kurochkina [1,2], Maksim A. Korolev [1,2] and Mariya A.Vorobyeva [1,*]**

**Supplementary materials**

**Table S1**. In vitro selection protocol

| | Step | Conditions |
|---|---|---|
| 1 | ssDNA library preparation | 1 nmol in 1× PBS, pH 7.4, 5 mM $MgCl_2$<br>95 °C – 5 min, ice - 5 min, room temperature – 15 min<br>Add 0.05% Tween 20, 100 µg/mL polyA, and 0.01 % HSA for the total volume of 200 µL<br>Incubate for 15 min at the room temperature |
| 2 | Negative selection | Transfer 15 µL of HisMag Sepharose Ni to a new tube, wash with 200 µL of PBSMT (1× PBS, pH 7.4, 5 mM $MgCl_2$, 0.05% Tween 20) 3 times<br>Add 200 µl of the library<br>Incubate 30 min at the room temperature and gentle mixing<br>Magnet – 1 min<br>Use the supernatant with an unbound DNA pool for the positive selection (Step 4) |
| 3 | DKK-1 immobilization | Transfer 15 µL of HisMag Sepharose Ni to a new tube, wash thrice with 200 µL of PBSMT<br>Add 100 µL of 20 µg/mL DKK-1 in 1× PBS<br>Incubate for 30 min at the room temperature and gentle mixing<br>Magnet – 1 min, remove the supernatant, wash the beads thrice with 200 µl of PBSMT |
| 4 | Positive selection | Add 200 µL of DNA pool from Step 2 to the beads from Step 3<br>Incubate for 60 min at the room temperature and gentle mixing |
| 5 | Elution | Add 15 µL of 20 mM Tris-HCl, pH 7.5, 100 mM imidazole to the beads from Step 4<br>10 min at room temperature and gentle mixing<br>Magnet – 1 min, collect the supernatant for amplification |

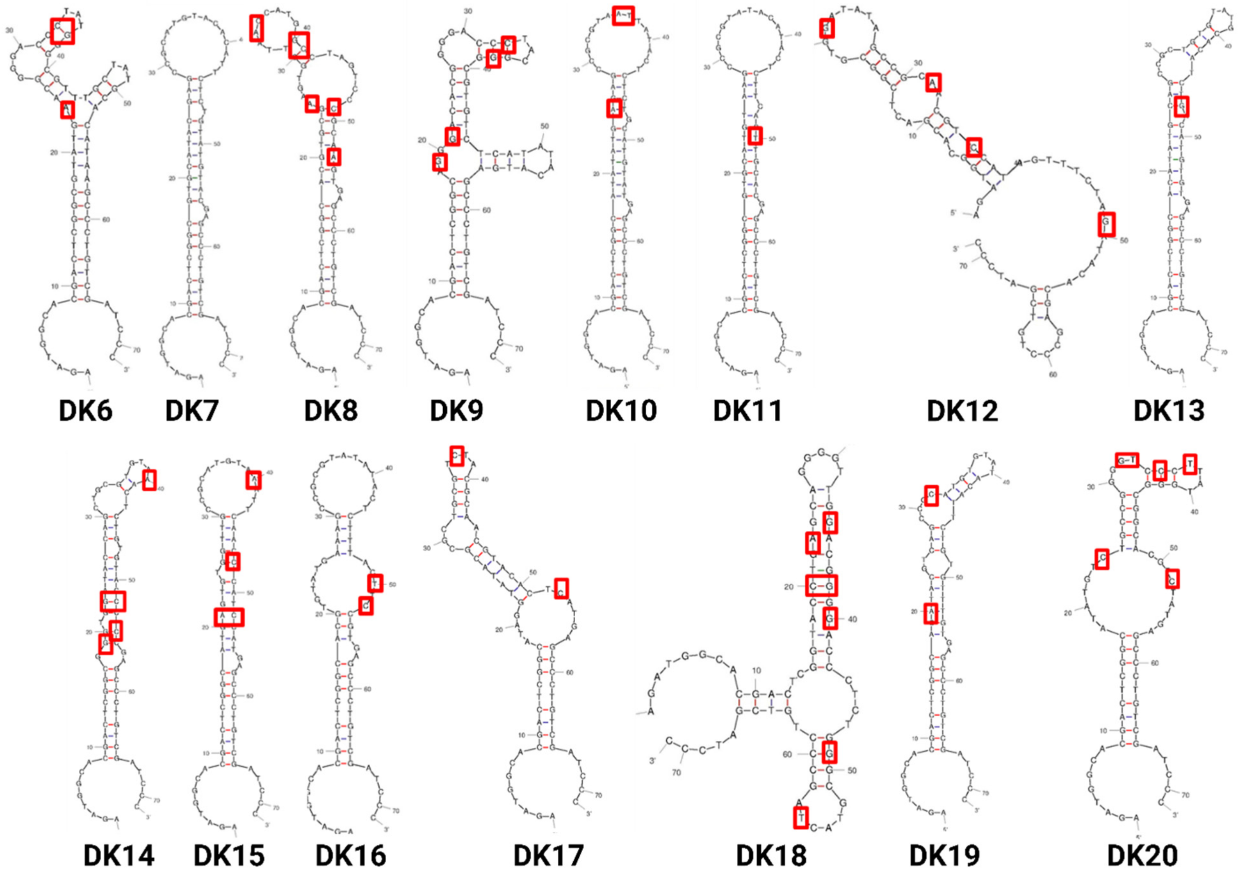


**Figure S1.** The most probable secondary structures of aptamers DK1-DK5 obtained with the mfold web server. Transversions in the pyrimidine-purine alternation pattern are highlighted in red.

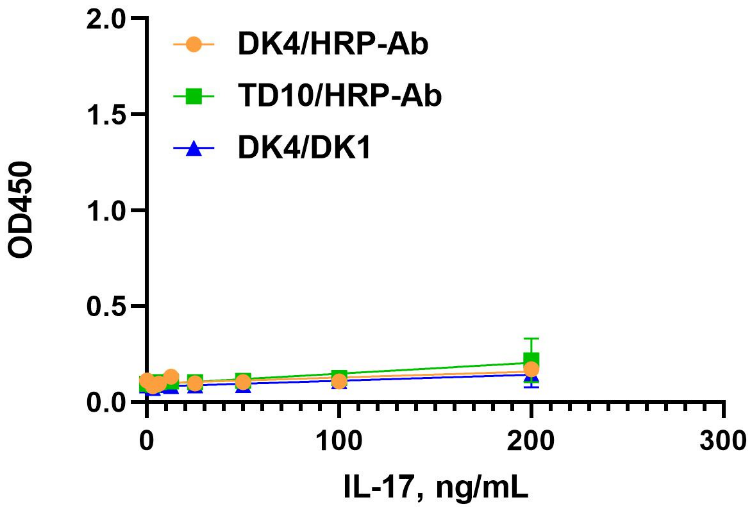


**Figure S2.** Dependencies of optical density from IL-17 concentration for aptamer/ antibody and aptamer/aptamer sandwich pairs. The GraphPad Prism 8.0.1 software package was used to plot the linear dependency of OD450 from IL-17 concentration. Each point represents the average value of two independent experiments.
